# 1862. Understanding Care Experiences Amongst Immigrant and Refugee Clients in a Ryan-White Funded HIV Clinic: Insights to Improve Culturally Competent Care

**DOI:** 10.1093/ofid/ofad500.1690

**Published:** 2023-11-27

**Authors:** Sirey Zhang, Antonia Altomare, Richard A Zuckerman

**Affiliations:** Geisel School of Medicine at Dartmouth, Hanover, New Hampshire; Dartmouth-Hitchcock Medical Center, Lebanon, New Hampshire; Dartmouth-Hitchcock Medical Center, Lebanon, New Hampshire

## Abstract

**Background:**

Immigrants and refugees receiving HIV care may have challenges accessing effective care due to their unique social determinants of health. In this pilot project, we gathered client and caregiver experiences to improve care systems for our immigrant and refugee clients.

**Methods:**

Participant observation and standardized ethnographic interviews were conducted with 18 clients and 4 caregivers in the HIV clinics at Dartmouth Health. Community support was determined by asking clients if there are family or other members of their immigrant community involved in supporting their care. Clients received gift cards for their time. The study was funded through the IDSA G.E.R.M. grant.

**Results:**

Clients encompassed the spectrum of documentation status and length of residence. Overall, clients seemed satisfied with their care; however, in addition to concerns of how their diagnoses impact documentation and employment status, clients cited fears about the impact on their community standing, with some clients refusing interpreters for fears of being identified. Seven of the 18 total clients reported poor community support. This group included the 2 clients who required more follow-up visits (9 and 4 visits per year) and the only client with an unsuppressed viral load. Congruently, caregivers perceived better adherence and participation when they were able to incorporate family and community support into the client’s care. Clients also expressed that it was important for them to incorporate their sense of self and community within the context of the predominant American culture.

**Conclusion:**

We found that for most of our clients, “culturally competent care” is a spectrum of integration of their indigenous and new cultural experiences. Care outcomes are improved when the clinic supports a client’s need to maintain community support, legal standing, and employment, all of which are key factors in immigrant/refugee success in a new country. However, fear of disclosure can be a barrier to establishing this support, requiring the clinic team to take an active role in building care support while ensuring privacy. Moving forward, we plan to restructure culturally competent care in our clinics in ways that include these important components.

**Disclosures:**

**All Authors**: No reported disclosures

